# Role of Calpain-1 in Neurogenesis

**DOI:** 10.3389/fmolb.2021.685938

**Published:** 2021-06-15

**Authors:** Michel Baudry, Wenyue Su, Jeffrey Seinfeld, Jiandong Sun, Xiaoning Bi

**Affiliations:** ^1^ Graduate College of Biomedical Sciences, Western University of Health Sciences, Pomona, CA, United States; ^2^ College of Osteopathic Medicine of the Pacific, Western University of Health Sciences, Pomona, CA, United States

**Keywords:** calpain, neurogenesis, dentate gyrus, gene expression, miRNA

## Abstract

While calpains have been implicated in neurogenesis for a long time, there is still little information regarding the specific contributions of various isoforms in this process. We took advantage of the availability of mutant mice with complete deletion of calpain-1 to analyze its contribution to neurogenesis. We first used the incorporation of BrdU in newly-generated cells in the subgranular zone of the dentate gyrus to determine the role of calpain-1 deletion in neuronal proliferation. Our results showed that the lack of calpain-1 decreased the rate of cell proliferation in adult hippocampus. As previously shown, it also decreased the long-term survival of newly-generated neurons. We also used data from previously reported RNA and miRNA sequencing analyses to identify differentially expressed genes in brain of calpain-1 knock-out mice related to cell division, cell migration, cell proliferation and cell survival. A number of differentially expressed genes were identified, which could play a significant role in the changes in neurogenesis in calpain-1 knock out mice. The results provide new information regarding the role of calpain-1 in neurogenesis and have implications for better understanding the pathologies associated with calpain-1 mutations in humans.

## Introduction

Mammalian adult neurogenesis was first observed in 1965, but the last two decades have seen considerable expansion of interest and effort to understand the biology of this phenomenon in mammals (Altman and Das, 1965; Hamburger, 1980; Spalding et al., 2013; Christian et al., 2014; Ernst et al., 2014). Adult neurogenesis is the process by which new neurons and glial cells are generated in the adult brain (Gage, 2000). It is both temporally and spatially regulated over the continuum of an organism‘s lifespan (Suh et al., 2009; Ming and Song, 2005; Christian et al., 2014). It was originally assumed that neurogenesis did not occur appreciably in mammals after the early postnatal period (Hamburger, 1980; Spalding et al., 2013). However, new evidence indicates that neurogenesis occurs throughout the lifetime (Ibrayeva and Bonaguidi, 2015). In adult humans, it is estimated that seven hundred new neurons are generated every week (Spalding et al., 2013; Ernst et al., 2014). The brain experiences the highest rate of neurogenesis in the immediate days following birth. As mammals transition to adulthood, the endogenous rate of proliferation begins to decline and the ability of newly generated neurons to survive and become incorporated into established neural networks decreases (Doetsch et al., 1999; Merkle et al., 2004; Ming and Song, 2005; Lepousez et al., 2013; Christian et al., 2014).

There are two discrete regions in the adult mammalian brain where neurogenesis is readily observable, the subventricular zone (SVZ) and the subgranular zone (SGZ) of the dentate gyrus (Altman and Das, 1965; Altman, 1969). In these two regions, stem cells undergo asymmetrical cell division wherein the stem cell self-renews, retaining its genetic and phenotypic identity, while the “daughter-cell” becomes a more differentiated neural progenitor cell (NPC) relative to its “mother” (Santos et al., 2012; Lepousez et al., 2013; Christian et al., 2014). The daughter NPCs then proliferate, differentiate, and migrate (Suh et al., 2009; Santos et al., 2012; Lepousez et al., 2013; Christian et al., 2014). NPCs generated in the SGZ and the SVZ can become neurons, glia, and other cell types, which migrate and integrate into neural networks in hippocampus and olfactory bulbs (Lepousez et al., 2013; Vadodaria and Gage, 2014).

Several studies have shown that calpains are actively involved in mammalian adult neurogenesis (Santos et al., 2012; Persson et al., 2013; Chung et al., 2015; Machado et al., 2015; Wang and Zhang, 2014). Furthermore, many studies provide additional support for a critical role for calpains in cell proliferation, survival, migration, and differentiation (Huttenlocher et al., 1997; Yajima and Kawashima, 2002; Moyen et al., 2004; Raynaud et al., 2008; Jessberger et al., 2009; Moudilou et al., 2010; Storr et al., 2011; Lade et al., 2012; Wang and Zhang, 2014; Chung et al., 2015; Liang et al., 2015). Calpains have been shown to be critical for cell proliferation and differentiation through their interactions with and cleavage of proteins involved in cell cycle regulation (Joy et al., 2006; Jessberger et al., 2009; Storr et al., 2011). In particular, the cyclin-dependent kinase 5 (cdk5) plays important roles in neural development (Kawauchi, 2014), and its activation requires the binding of its regulatory subunit, p35. Calpain cleaves p35 into p25, which increases cdk5 activity and changes its substrate selectivity and subcellular localization. Calpains also play an important role for cell survival during postnatal development, as demonstrated by the loss of many cells during development in *Capn1*-KO mice and the embryological lethality in *Capns1* ^−/−^ and *Capn2* ^−/−^ mice (Arthur et al., 2000; Zimmerman et al., 2000; Dutt et al., 2006; Takano et al., 2011; Amini et al., 2013; Wang et al., 2016). In addition, calpains could regulate distinct steps in neuronal differentiation by regulation of epigenetic DNA modifications, cell cycle progression, migration, and response to extracellular trophic signals (Moors et al., 2007; Suh et al., 2009; Santos et al., 2012; Wang and Zhang, 2014). Calpains have been shown to cleave several proteins involved in the differentiation of several cell types, including Ten-eleven translocation (Tet) proteins, ß-catenin and CCAAT/enhancer-binding protein (C/EBP) β (Moyen et al., 2004; Abe and Takeichi, 2007; Zhang et al., 2012; Wang and Zhang, 2014).

It has been difficult to evaluate the specific roles of the two major calpain isoforms in the brain, calpain-1 and calpain-2, in neurogenesis, due to the lack of selective inhibitor and the lack of mutant mice with selective down-regulation of calpain-1 or calpain-2. During neuronal differentiation, calpain-1 is highly expressed in neural stem cells (NSCs) and immature neural progenitor cells (NPCs), while the expression of calpain-2 increases with continuing neuronal differentiation (Wu et al., 2010; Santos et al., 2012; Machado et al., 2015). Differential expression of calpain isoforms has also been observed in many differentiating cell types, including myocytes, hepatocytes, osteoblasts, and adipocytes (Yajima and Kawashima, 2002; Moyen et al., 2004; Lade et al., 2012). Similar to what is observed in neuronal differentiation, high levels of calpain-1 are observed in immature progenitors, while calpain-2 levels increase with continuing states of differentiation (Yajima and Kawashima, 2002; Dedieu et al., 2003; Honda et al., 2008; Wu et al., 2010; Lade et al., 2012; Wamstad et al., 2012). The present studies took advantage of the availability of calpain-1 knock-out (C1KO) mice to more precisely analyze the role of calpain-1 in neurogenesis and the potential mechanisms by which calpain-1 could regulate neuronal division, differentiation, proliferation, migration and survival.

## Materials and Methods

### Animals

Animal use in all experiments followed the National Institutes of Health (NIH) guidelines and all protocols were approved by the Institution Animal Care and Use Committee of Western University of Health Sciences.

### 5-Bromo-2-Deoxyuridine Labeling

Three-month-old C57/Bl6 (wild-type (WT) and *Capn1*-KO on a C57/Bl6 background) male mice were kept under standard laboratory conditions with a 12:12 h light/dark cycle. Mice were injected intraperitoneally (i.p.) with 5-bromo-2-deoxyuridine (BrdU) (50 mg/kg) every 8 h for 3 days, a total of nine injections. Brains were collected following transcardial perfusion after 3 or 30 days from the first BrdU injection. They were immersed in 4% PFA for 24–48 h before transfer to a 30% sucrose solution for 48 h. Preparation of 30 μm-thick tissue sections was performed by embedding brains in 2% agarose and sectioning them with a Leica VT000 S vibratome. Sections were rinsed (3 × 5 min) in 0.01 M phosphate buffer saline containing 0.3% Triton X-100 (PBS-TX). For BrdU immunostaining, sections were pre-treated with 2 M hydrochloride and Triton-X 100 at 37°C for 45 min followed by 10 min washing in borate buffer (pH 8.5) in order to denaturate DNA. Sections were incubated in anti-NeuN (abcam ab104224, ab177487), anti–BrdU (SCBT sc-32323), or anti–Ki67 (abcam ab16667) antibodies for 48 h at 4°C followed by incubation in secondary antibodies (Life technologies Alexa-fluor) for 2 h at room temperature. One tenth of the collected sections were stained and imaged with a Nikon Te-2000 confocal microscope at ×20 magnification. Images were then analyzed using Macro enabled Image-J open source software. All BrdU- or Ki67- positive cells in a 500 × 500 µm area were counted using ImageJ. Five sections from each mouse at various levels of the hippocampus or the olfactory bulbs were analyzed and the data averaged for this mouse. Quantitative data were normalized to percent of control.

### P35-P25 Conversion

Mice were deeply anesthetized using gaseous isoflurane and decapitated. The whole brain was removed, and hippocampus dissected. Tissue was homogenized in radioimmunoprecipitation assay (RIPA) buffer (10 mM Tris, pH 8, 140 mM NaCl, 1 mM EDTA, 0.5 mM EGTA, 1% NP-40, 0.5% sodium deoxycholate, and 0.1% SDS). Protein concentrations were determined with a BCA protein assay kit (Pierce). Western blots were performed according to published protocols (Sun et al., 2015). Briefly, proteins were separated by SDS-PAGE and transferred onto a PVDF membrane (Millipore). After blocking with 3% BSA for 1 h, membranes were incubated with p35 antibodies (1:500; catalog #sc-820; Santa Cruz Biotechnology) overnight at 4°C followed by incubation with IRDye secondary antibodies for 2 h at room temperature. Antibody binding was detected with the Odyssey® imaging system (LI-COR Biosciences), and Western blots were analyzed with the Image Studio Software.

### miRNA Target Prediction

To explore neurogenesis-related DE miRNAs found in the miRNA-seq results, the targets of DE miRNAs were provided from the miRTarBase database via the MIENTURNET (MicroRNA Enrichment TURend NETwork) tool (Licursi et al., 2019), and then filter on the basis of a *p*-value <0.05.

### Gene Ontology Analysis

DAVID (https://david.ncifcrf.gov/) and GENEONTOLOGY (http://geneontology.org/) were used for extraction of the genes belonging to the “neurogenesis” (GO:0022008) GO terms. These programs may utilize different dataset for assessing GO (Gene Ontology) enrichment and therefore may complement each other. We used a combined list as candidate genes related to neurogenesis.

### Statistical Analyses

Data are generally presented as means ± SD or means ± SEM. In all experiments, only two groups were compared, thus, a two-tail *t*-test was used for determining statistical significance with no correction of variability of standard deviation. *p* values less than 0.05 were considered statistically significant. Due to the small sample size, no attempt was made to test the normality of data.

## Results

### Role of Calpain-1 in Neurogenesis in the Dentate Gyrus

**FIGURE 1:**
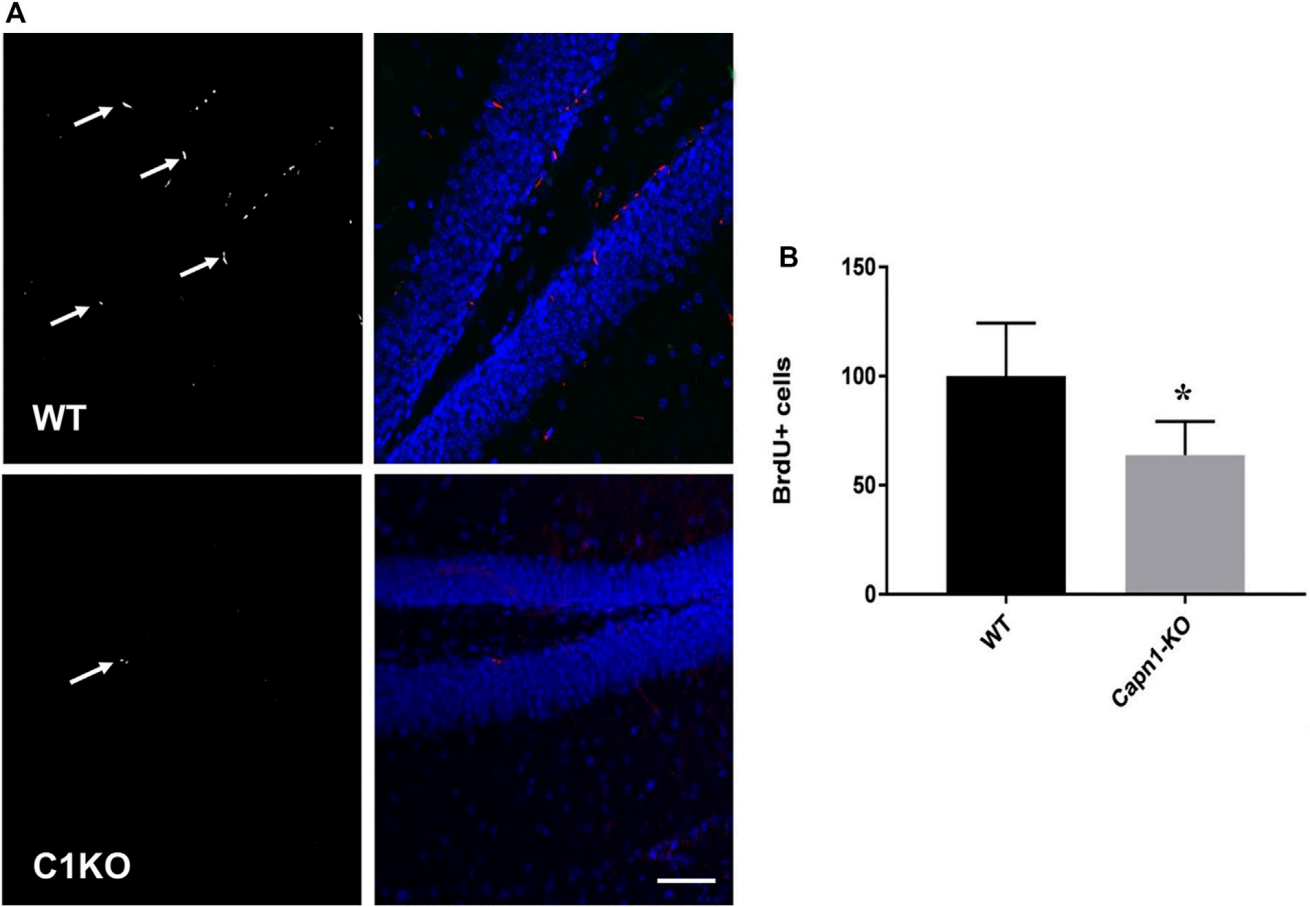
BrdU+ cells in the dentate gyrus of WT and C1KO mice. WT and C1KO mice were injected i.p. with 5-bromo-2-deoxyuridine (BrdU) (50 mg/kg) every 8 h for 3 days, for a total of nine injections. Brains were collected following transcardial perfusion 3 days after the first BrdU injection. Brains were sectioned and sections were processed for immunolabeling with antibodies against BrdU **(A)**, left panels, arrows) and NeuN **(A)**, right panels, blue). Numbers of BrdU+ cells were quantified as indicated in the *Materials and Methods* section **(B)**. Sections were counterstained with DAPI (blue). Five sections per mouse were quantified and 5 WT and C1KO mice were analyzed. Results are expressed as percent of the average number of cells in the WT mice and are Means ± SEM of 5 mice per group. *p < 0.01, Student’s t-test. Scale bar: 50 µm.

**FIGURE 2:**
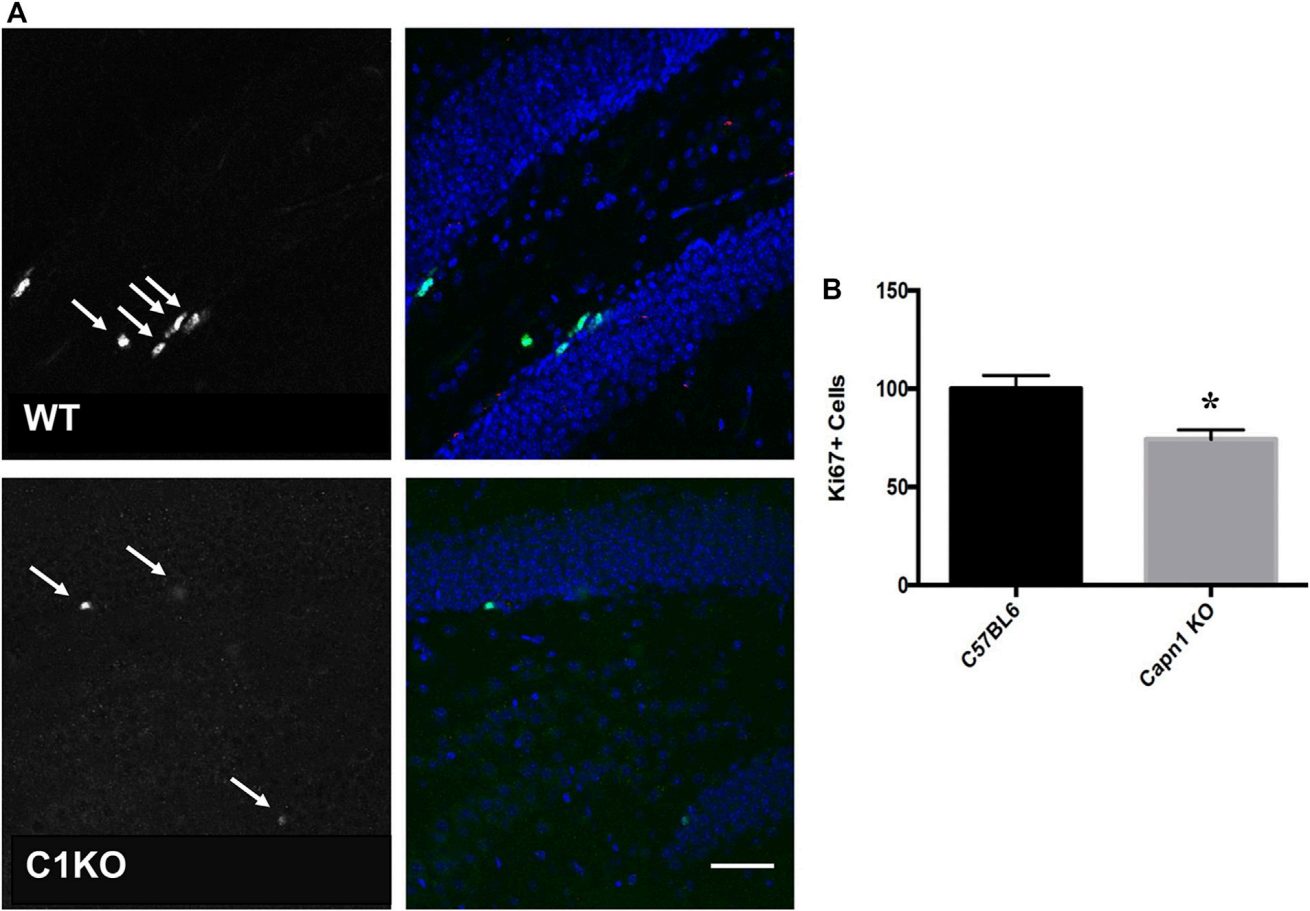
Ki67+ cells in the dentate gyrus of WT and C1KO mice. Adjacent sections from the ones used for the BrdU analysis were used for immunolabeling with antibodies against Ki67 **(A)**, left panels, arrows) and NeuN **(A)**, right panels, green). Numbers of Ki67+ cells were quantified as indicated in the *Materials and Methods* section **(B)**. Sections were counterstained with DAPI (blue). Five sections per mouse were quantified and 5 WT and C1KO mice were analyzed. Results are expressed as percent of the average number of cells in the WT mice and are Means ± SEM of 5 mice per group. *p < 0.01, Student’s t-test. Scale bar: 50 µm.

**FIGURE 3:**
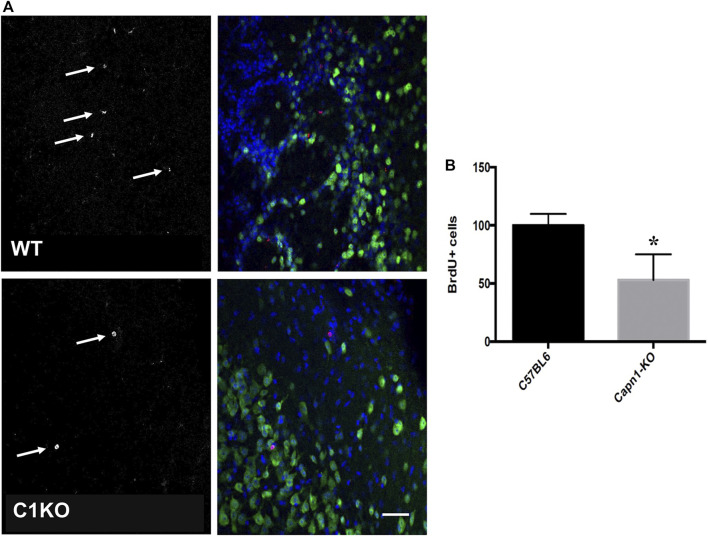
BrdU+ cells in the olfactory bulbs of WT and C1KO mice. WT and C1KO mice were injected with 5-bromo-2-deoxyuridine (BrdU) (50 mg/kg) every 8 h for 3 days, a total of nine injections. Brains were collected following transcardial perfusion 30 days after the first BrdU injection. Brains were sectioned and sections were processed for immunolabeling with antibodies against BrdU **(A)**, left panels arrows, red in left panels) and NeuN **(A)**, right panels, green). Sections were counterstained with DAPI (blue). Numbers of BrdU+ cells were quantified as indicated in the *Materials and Methods* section **(B)**. Five sections per mouse were quantified and 5 WT and C1KO mice were analyzed. Results are expressed as percent of the average number of cells in the WT mice and are Means ± SEM of 5 mice per group. *p < 0.01, Student’s t-test. Scale bar: 50 µm.

Evidence suggests that calpain-1 is important for cell proliferation and self-renewal of NSCs (Santos et al., 2012; Wang and Zhang, 2014). We investigated the rate of proliferation in the dentate gyrus of C1KO mice using the canonical proliferation expression marker Ki67, and BrdU-incorporation. We found a >25% decrease for both BrdU^+^ and Ki67^**+**^ cells (*n* = 5–6 per group, *p < 0.05 WT vs. C1KO) in the DG of C1KO mice (Figures 1, 2). The similarities between the changes in BrdU^+^ and Ki67^**+**^ cells suggest that most BrdU^+^ cells are neurons. This result suggests that calpain-1 activity is important for the proliferation of newly-born cells in the dentate gyrus. We also analyzed the migration/survival of newly-born cells by determining the number of BrdU^+^ cells in the olfactory bulbs 30 days after injection (Figure 3). The number of BrdU^+^ cells was significantly decreased in C1KO mice, as compared to WT mice.

**FIGURE 4:**
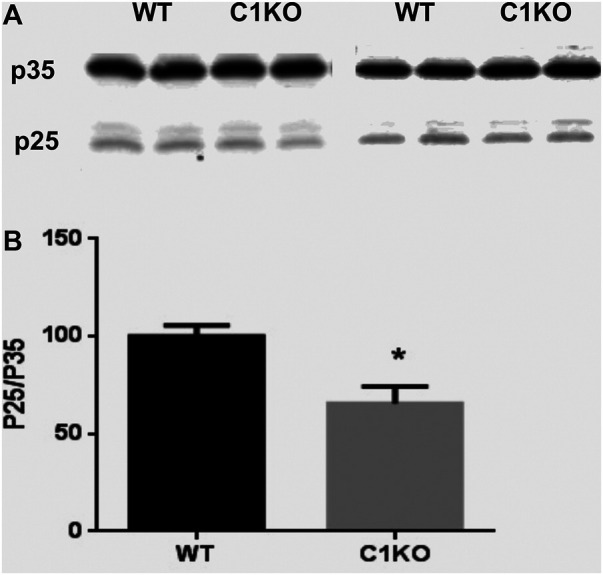
Levels of p35 and p25 in the hippocampus of WT and C1KO mice. WT and C1KO mice were sacrificed, and their hippocampi collected. They were homogenized and aliquots of the homogenates processed for immunoblotting with p35 antibodies **(A)**. **(B)** Quantification analysis. Ratios of p25/p35 were calculated and results were expressed as percent of the averaged value in WT mice and represent Means ± SEM of 4 animals. *p < 0.01, Student’s t-test.

Cdk5 plays important roles in neuronal development and survival (Kawauchi, 2014), and it has been shown that calpain-mediated truncation of p35–p25 promotes neural development and differentiation (Jessberger et al., 2009; Shu et al., 2018). We therefore evaluated the levels of p35 and p25 in hippocampus of WT and C1KO mice (Figure 4). While p35 levels were not significantly different between WT and C1KO mice, the ratio p25/p35 was significantly decreased in hippocampus from C1KO mice as compared to WT mice. These results suggest that p35 is tonically cleaved to p25 by calpain-1 and that the lack of calpain-1 results in decreased cleavage, which would be associated with reduced cdk5 activity in the C1KO mice.

### Changes in Neurogenesis Genes and Gene Networks in Calpain-1 Knock-Out Mice

We recently performed RNA-Seq analysis to compare gene expression in brains of WT and C1KO mice (Su et al., 2020). We reanalyzed the data to identify which genes related to neurogenesis were differentially expressed in brains of these mice (Supplementary Table S1). We identified 60 genes that were differentially expressed (DE), with nine genes upregulated and 51 downregulated. We categorized these genes into four clusters related to cell cycle, cell migration, cell division and cell death (Table 1). Interestingly, one of the genes significantly down-regulated in the brains of C1KO mice is Nr4a1, which we previously validated in our RNA-Seq analysis by RT-qPCR and showed that the protein was also down-regulated in hippocampus. Nr4a1 is involved in many functions including apoptosis, and we found that it colocalized with doublecortin in the dentate gyrus and was present in newly-born neurons (Su et al., 2020). Nr4a3 has also been previously identified as playing a role in hippocampal neurogenesis (Ashbrook et al., 2014) and is significantly downregulated in brains of C1KO mice.

**TABLE 1:** GO analysis of differentially expressed neurogenesis-related genes in brains of C1KO, as compared to WT mice.

GO term	Gene symbol
Cell cycle	Top2b; Nr4a3; App; Ank3
Cell migration	Sorl1; Top2b; D130043K22Rik; Nf1; Ndn; Mapt; Reln; Fn1; Slit1; Fat3; Trio; Plxna4; Sema4d; Cx3cl1
Cell division	Etv5; Ank3
Cell death	Nr4a1; Nr4a3; App; Nf1; Xbp1; Mapt; Ywhaz; Tnfrsf21; Grin2a; Fn1; Hsp90ab1

DE genes listed in Supplementary Table S1 were separated based on the listed GO terms.

**FIGURE 5:**
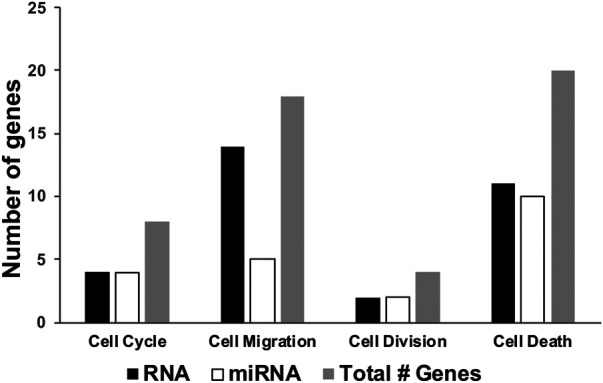
Number of differentially expressed genes related to neurogenesis in brains of C1KO mice as compared to WT mice. The number of differentially expressed genes, based on RNA sequencing or miRNA-targeted or both, were compiled for cell cycle, cell migration, cell division or cell death.

**FIGURE 6:**
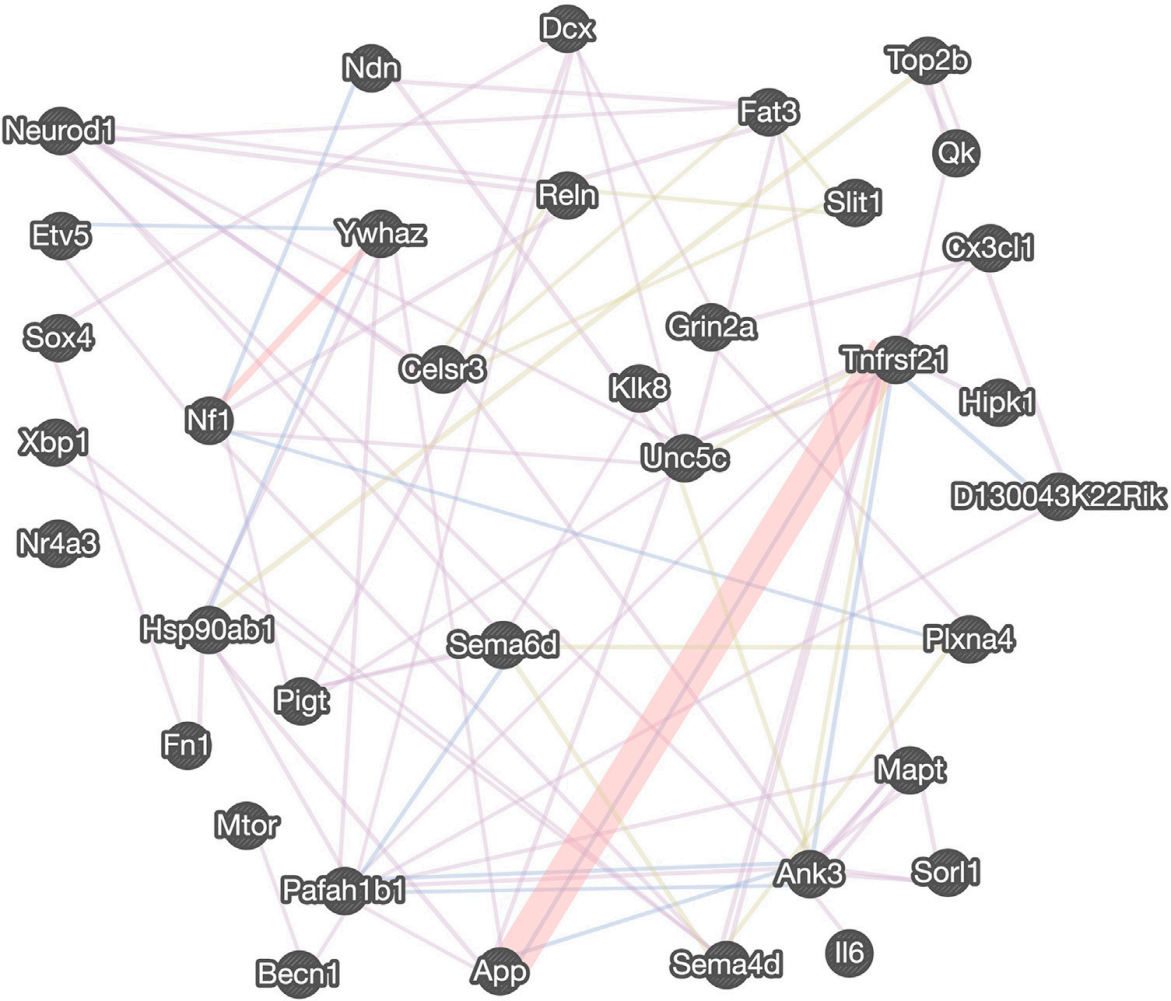
Gene interaction network regulated by calpain-1 for cell cycle, cell division and cell survival. The gene interaction network was derived from the GeneMANIA tool using the neurogenesis-related DE genes and targets of DE miRNAs, assigned to “cell cycle,” “cell migration,” “cell division” and “cell death” GO terms. The distinct colors of the network line indicate the dataset applied: co-expression (purple), physical interactions (red), co-localization (blue), shared protein domains (light green). Five transcription factors are listed on the left.

We also recently analyzed changes in miRNAs in brains of C1KO mice, as compared to WT mice (Su et al., 2021). Again, we reanalyzed the data to identify those miRNAs related to neurogenesis (Table 2). We identified 24 miRNAs that were differentially expressed in brains of C1KO mice as compared to WT mice, with three upregulated and 21 downregulated mi-RNAs. We then analyzed the target genes for the DE miRNAs and identified those involved in cell cycle, cell migration, cell division and cell death (Table 3). Surprisingly, there was no overlap between the DE RNAs and the genes targeted by the DE miRNAs. To further analyze the important target genes from the DE miRNAs, we selected those for which the miRNAs exhibited the largest changes in expression between WT and C1KO mice (Table 4). Five genes appeared to be regulated by multiple miRNAs, including Map2 (microtubule-associated protein 2), Mtor (mechanistic target of rapamycin kinase), Qk (quaking, KH domain containing RNA binding), Dcx (doublecortin) and Zeb1 (zinc finger E-box binding homeobox1). Overall, there were 50 genes regulated by calpain-1 involved in cell cycle, cell migration, cell division and cell death (Figure 5). We used the GeneMANIA tool to build a gene interaction network using all the genes identified in the cell cycle, cell migration, cell division and cell death GO terms (Figure 6). A number of nodes were clearly revealed by this analysis, and the genes in these nodes are indeed related to cell death, growth cone and brain development.

**TABLE 2:** Differentially expressed miRNAs related to neurogenesis in brains of C1KO, as compared to WT mice.

miRNA	WT ave.log2.cpm	SEM	C1KO ave.log2.cpm	SEM	%WT	*p* value
mmu-miR-29a-3p	14.981	0.006	14.807	0.025	84	0.024
mmu-miR-99b-5p	12.645	0.012	12.756	0.026	112	0.004
mmu-miR-379-5p	11.712	0.016	11.851	0.024	115	0.015
mmu-miR-384-3p	11.522	0.031	11.348	0.036	84	0.010
mmu-miR-30e-5p	11.267	0.024	11.088	0.029	84	0.009
mmu-miR-101a3p	10.119	0.026	9.808	0.061	73	0.008
mmu-miR-146a5p	9.792	0.078	9.545	0.006	78	0.001
mmu-miR-29c-3p	9.506	0.014	9.250	0.006	77	0.004
mmu-miR-10b-5p	9.474	0.253	8.663	0.261	44	0.027
mmu-miR-370-3p	8.902	0.018	9.003	0.024	111	0.011
mmu-miR-10a-5p	8.822	0.144	8.380	0.114	64	0.018
mmu-miR-136-5p	8.421	0.033	8.287	0.021	87	0.011
mmu-miR-451a	8.313	0.209	8.076	0.117	79	0.008
mmu-miR-144-3p	6.806	0.243	6.453	0.116	70	0.002
mmu-miR-325-3p	6.202	0.032	6.042	0.072	85	0.024
mmu-miR-362-3p	5.915	0.021	5.660	0.075	78	0.011
mmu-miR-582-5p	5.816	0.017	5.553	0.058	77	0.011
mmu-miR-34b-5p	5.411	0.081	5.182	0.043	80	0.006
mmu-miR-200a3p	5.342	0.824	4.567	0.397	46	0.020
mmu-miR-142a3p	4.967	0.056	4.734	0.078	79	0.012
mmu-miR-142a5p	4.435	0.059	4.112	0.089	72	0.007
mmu-miR-429-3p	4.206	0.548	3.512	0.263	50	0.006
mmu-miR-200b3p	3.846	0.579	3.169	0.332	51	0.013
mmu-miR-19a-3p	3.536	0.112	3.113	0.069	66	0.001

Results are expressed as ave.log2.cpm and represent Means and S.E.M. of 3 mice per group.

**TABLE 3:** Target genes of the DE miRNAs in C1KO mice, as compared to WT mice, and corresponding GO terms.

Cell cycle	Cell migration	Cell division	Cell death
Neurod1	Sema6d	Pafah1b1	Klk8
Pafah1b1	Dcx	Becn1	Qk
Becn1	Unc5c		Becn1
Sox4	Pafah1b1		Sox4
	Celsr3		Neurod1
			Pigt
			Il6
			Hipk1
			Unc5c
			Mtor

Target genes of the DE-miRNAs were separated based on the listed GO terms.

**TABLE 4:** Predicted targets of selected neurogenesis-related DE miRNAs.

miRNAs	Gene1	Gene2	Gene3	Gene4	Gene5	Gene6	Gene7
↑mmu-miR-99b-5p	Mtor						
↑mmu-miR-370-3p	Mtor	Qk					
↑mmu-miR-379-5p	Slc11a2						
↓mmu-miR-10b-5p	Hook3	Mfn1	Nrcam	Crkl	Mdga2	Bloc1s3	Kif3a
↓mmu-miR-429-3p	Map2	Rassf10	Zeb1				
↓mmu-miR-200b-3p	Map2	Pafah1b1	Rassf10	Zeb1	Dcx		
↓mmu-miR-200a-3p	Dcx	Qk	Pafah1b1	Zeb1			

↑ refers to up-regulated miRNAs, ↓ refers to the three down-regulated miRNAs with the largest decreases.

## Discussion

Our study sought to investigate the role of the calpain specific isoform, calpain-1, in proliferation, survival and migration of adult neural stem cells *in vivo*. We found that the survival of newborn cells in the OB of adult C1KO mice labeled with BrdU was significantly reduced, as compared to that in WT mice. No significant difference was found in the amount of BrdU^+^ cells migrating in the rostral migratory stream (RMS) (data not shown). The similarities on the changes in BrdU^+^ and Ki67^+^ cells suggest that these cells are newly-born neurons. This result suggests that survival of newly-born neurons is dependent on calpain-1 activity, which is in good agreement with the notion that calpain-1 is neuroprotective (Wang et al., 2013; Wang et al., 2016).

We also observed that proliferation of newly-born cells in the dentate gyrus was decreased in C1KO mice. The decrease in proliferation we observed was comparable to that found in a previous study on neural stem cells (NSC) proliferation in the dentate gyrus in the *Capns1*-KO mice (Santos et al., 2012; Amini et al., 2013), in which the activities of both calpain-1 and -2 were disrupted. To our knowledge, our study is the first to investigate the effect of the loss of function of a specific calpain isoform in adult neurogenesis *in vivo*. It remains to be investigated whether deletion of calpain-2 would have a similar effect on proliferation of NSCs in the dentate gyrus. Studies investigating proliferation in other cell systems have provided ample evidence for a role for calpain-1 (Moudilou et al., 2010; Storr et al., 2011; Santos et al., 2012).

We found no significant difference in the number of BrdU^**+**^ cells in the RMS between C1KO and WT mice. This result suggests that the migration of differentiating NSCs from the SVZ is not critically dependent on calpain-1. Previous reports examining the differential role of calpain-1 and calpain-2 in adult neurogenesis *in vitro* have suggested that calpain-1 activity is important for NSC proliferation and self-renewal, while calpain-2 activity has been shown in many different cell types to be important for cell migration (Moyen et al., 2004; Santos et al., 2012). Investigation of the molecular mechanisms regulating differentiation of several cell types (embryonic stem cells, induced pluripotent cells, adipose derived stromal/cells, myocytes) has demonstrated that calpain-2 is critically important for migration of differentiating cells (Dedieu et al., 2003; Raynaud et al., 2008). Our evidence that calpain-1 deletion does not negatively affect the migration of NSCs from the SVZ, is consistent with these previous studies.

Analysis of genes and miRNAs differentially expressed in C1KO mice offers several potential mechanisms responsible for the changes in cell proliferation and survival we observed between WT and C1KO mice. The expression of a significant number of genes involved in cell cycle, cell division, cell differentiation and cell survival is altered in brain of C1KO mice. Likewise, the expression of many miRNAs targeting genes involved in these processes are altered. At least 50 genes involved in these cellular processes are differentially expressed in brain of C1KO mice. The gene network analysis provided a number of critical nodes regulating cell survival and cell proliferation, which could be responsible for the various effects we observed in C1KO mice. This is in particular the case for Grin2a, which encodes the NR2A subunit of the NMDA receptors and has been shown to be upstream of calpain-1 and involved in neuroprotection (Wang et al., 2013). Several DE genes are also involved in growth cone motility, including Sema6d, Unc5c, and Ank3. In addition, calpain-1 has been shown to regulate the differentiation of mouse embryonic stem cells to neuronal cells through degradation of ten-eleven translocation (TET) proteins, which regulate DNA demethylation (Wang and Zhang, 2014). Calpain-1 has also been shown to regulate developmental organogenesis via N-terminal truncation and activation of β-catenin, a family member of the Wnt signaling pathway (Lade et al., 2012) and through the STAT3/HIF-1α/VEGF pathway (Wu et al., 2018). Loss of regulation of distinct TFs and epigenetic modifications of DNA are possible molecular mechanisms underlying the reduced proliferation observed in the dentate gyrus of C1KO mice. As we used the whole brain and not the dentate gyrus for the mRNA and miRNA analysis, it is possible that we missed some genes and miRNAs with low levels of expression. We also found that ratios of p25/p35 are decreased in hippocampus of C1KO mice as compared to WT mice, suggesting that cdk5 is less active in the C1KO mice, which could participate in impaired development in these mice. This could also play a role in the learning and memory impairment we previously reported in these mice (Zhu et al., 2015), as cdk5 also plays a crucial role in spatial learning (Mishiba et al., 2014).

In conclusion, our results clearly demonstrate that calpain-1 plays an important role in neurogenesis in the mouse brain, which could account for several of the phenotypic alterations previously reported, including cerebellar ataxia, impaired synaptic plasticity and learning, and increased susceptibility to brain injury. These results underscore the needs to further evaluate human subjects with calpain-1 mutations for possible defects in neurogenesis.

## Data Availability

The original contributions presented in the study are included in the article/Supplementary Material, further inquiries can be directed to the corresponding author.
