# Supplementary material for: Role of Calpain-1 in Neurogenesis
Source: Front Mol Biosci. 2021 Jun 15;8:685938. doi: 10.3389/fmolb.2021.685938 (PMC8239220; doi:10.3389/fmolb.2021.685938)
Supplement: Supplementary file 1 [file Table1.DOCX]

*Supplementary Material*

**Supplementary Table**

**Table S1. DE RNAs related to neurogenesis in brain of C1KO mice**

| **Gene** | **WT Exp Level** | **SEM** | **C1KO Exp Level** | **SEM** | **% WT** | **p value** |
| --- | --- | --- | --- | --- | --- | --- |
| Kif5a | 23495 | 254 | 21390 | 270 | 91 | 0.0026224 |
| App | 18733 | 248 | 16390 | 258 | 87 | 0.0000076 |
| Hsp90ab1 | 15207 | 143 | 12881 | 447 | 85 | 0.0000008 |
| Psap | 14131 | 206 | 12796 | 124 | 91 | 0.0026487 |
| Map1a | 12648 | 574 | 10279 | 298 | 81 | 0.0000017 |
| Map1b | 11608 | 351 | 9112 | 457 | 78 | 0.0000000 |
| Ncdn | 11114 | 326 | 10044 | 198 | 90 | 0.0194374 |
| Ywhaz | 7589 | 150 | 6922 | 149 | 91 | 0.0323130 |
| Atp2b2 | 7446 | 310 | 6181 | 146 | 83 | 0.0000155 |
| Slc12a5 | 6632 | 140 | 5937 | 212 | 90 | 0.0171658 |
| Dst | 6261 | 221 | 5187 | 186 | 83 | 0.0000167 |
| Mapt | 5285 | 96 | 4672 | 58 | 88 | 0.0014698 |
| Ank3 | 5275 | 134 | 4373 | 77 | 83 | 0.0000006 |
| Sorl1 | 5082 | 166 | 4336 | 196 | 85 | 0.0013060 |
| Cx3cl1 | 5017 | 189 | 4348 | 156 | 87 | 0.0046268 |
| Mycbp2 | 4556 | 40 | 3903 | 186 | 86 | 0.0006720 |
| Nfasc | 3985 | 177 | 3383 | 184 | 85 | 0.0053742 |
| Herc1 | 3097 | 27 | 2557 | 141 | 83 | 0.0000967 |
| Ina | 2658 | 123 | 2272 | 15 | 85 | 0.0046268 |
| Nrep | 2291 | 64 | 2976 | 221 | 130 | 0.0000035 |
| Trio | 2277 | 58 | 2022 | 53 | 89 | 0.0406382 |
| Dner | 2238 | 23 | 1904 | 93 | 85 | 0.0024737 |
| Fry | 2028 | 61 | 1582 | 52 | 78 | 0.0000006 |
| Prickle2 | 1809 | 29 | 1534 | 81 | 85 | 0.0064201 |
| Ndn | 1671 | 57 | 2444 | 80 | 146 | 0.0000000 |
| Bend6 | 1530 | 28 | 1772 | 53 | 116 | 0.0101978 |
| Igsf9b | 1526 | 67 | 1294 | 71 | 85 | 0.0249177 |
| Kcna1 | 1525 | 52 | 1303 | 27 | 85 | 0.0120112 |
| Reln | 1490 | 31 | 1298 | 60 | 87 | 0.0418060 |
| Grin2a | 1428 | 49 | 1193 | 37 | 84 | 0.0035526 |
| Ppp1cc | 1420 | 37 | 1680 | 113 | 118 | 0.0175850 |
| L1cam | 1388 | 64 | 1113 | 71 | 80 | 0.0019859 |
| Top2b | 1337 | 30 | 1526 | 58 | 114 | 0.0474263 |
| Tenm4 | 1325 | 31 | 1122 | 53 | 85 | 0.0141889 |
| Slc9a6 | 1303 | 20 | 1555 | 58 | 119 | 0.0018538 |
| Map6 | 1207 | 38 | 1038 | 30 | 86 | 0.0339884 |
| Camsap1 | 1204 | 45 | 1031 | 8 | 86 | 0.0259475 |
| Plxna4 | 1177 | 19 | 1013 | 13 | 86 | 0.0224056 |
| Nf1 | 1158 | 37 | 953 | 73 | 82 | 0.0142320 |
| Fat3 | 1077 | 25 | 874 | 42 | 81 | 0.0021577 |
| Etv5 | 1050 | 52 | 886 | 37 | 84 | 0.0361907 |
| Tenm1 | 1036 | 23 | 847 | 65 | 82 | 0.0113396 |
| Tnfrsf21 | 1035 | 25 | 889 | 3 | 86 | 0.0323006 |
| Bhlhe40 | 1012 | 73 | 798 | 50 | 79 | 0.0062243 |
| Fryl | 970 | 28 | 821 | 13 | 85 | 0.0228365 |
| Sema4d | 955 | 22 | 808 | 15 | 85 | 0.0211731 |
| Itpka | 886 | 39 | 725 | 24 | 82 | 0.0099967 |
| Atxn1 | 848 | 32 | 664 | 10 | 78 | 0.0005503 |
| Xbp1 | 839 | 41 | 649 | 7 | 77 | 0.0004342 |
| Fn1 | 727 | 11 | 589 | 12 | 81 | 0.0062243 |
| Myrf | 702 | 32 | 570 | 16 | 81 | 0.0157775 |
| Slit1 | 625 | 23 | 489 | 43 | 78 | 0.0129449 |
| Bex1 | 616 | 12 | 466 | 17 | 76 | 0.0004508 |
| Nr4a3 | 501 | 30 | 331 | 7 | 66 | 0.0000031 |
| Pak6 | 496 | 27 | 606 | 22 | 122 | 0.0406382 |
| Fabp7 | 434 | 9 | 621 | 32 | 143 | 0.0000035 |
| D130043K22Rik | 422 | 16 | 325 | 15 | 77 | 0.0132639 |
| Dynlt1 | 36 | 5 | 108 | 15 | 301 | NA |
